# Uterine Cesarean Scar Tissue—An Immunohistochemical Study

**DOI:** 10.3390/medicina60040651

**Published:** 2024-04-18

**Authors:** Maciej Ziętek, Małgorzata Świątkowska-Feund, Sylwester Ciećwież, Tomasz Machałowski, Małgorzata Szczuko

**Affiliations:** 1 Department of Perinatology, Obstetrics and Gynecology, Pomeranian Medical University, 71-010 Police, Poland; maciej.zietek@pum.edu.pl (M.Z.); sylwester.ciecwiez@pum.edu.pl (S.C.); tomasz.machalowski@poczta.onet.pl (T.M.); 2 The Academy of Applied Medical and Social Sciences, 82-300 Elbląg, Poland; mal_swi@yahoo.com; 3 Department of Human Nutrition and Metabolomics, Pomeranian Medical University, 70-204 Szczecin, Poland

**Keywords:** uterine cesarean scar, immunohistochemistry, cesarean section, cluster of differentiation 31 antigen

## Abstract

*Background and Objectives*: Wound healing encompasses a multitude of factors and entails the establishment of interactions among components of the basement membrane. The quantification of particle concentrations can serve as valuable biomarkers for assessing biomechanical muscle properties. The objective of this study was to examine the immunoexpression and immunoconcentration of myometrial collagen type VI, elastin, alpha-smooth muscle actin, and smooth muscle myosin heavy chain, as well as the expression of platelets and clusters of differentiation 31 in the uterine scar following a cesarean section (CS). *Materials and Methods*: A total of 177 biopsies were procured from a cohort of pregnant women who were healthy, specifically during the surgical procedure of CS. The participants were categorized into seven distinct groups. Group 1 consisted of primiparas, with a total of 52 individuals. The subsequent groups were organized based on the duration of time that had elapsed since their previous CS. The analysis focused on the immunoexpression and immunoconcentration of the particles listed. *Results*: No significant variations were observed in the myometrial immunoconcentration of collagen type VI, elastin, smooth muscle myosin, and endothelial cell cluster of differentiation 31 among the analyzed groups. The concentration of alpha-smooth muscle actin in the myometrium was found to be significantly higher in patients who underwent CS within a period of less than 2 years since their previous CS, compared to those with a longer interval between procedures. *Conclusions*: Our findings indicate that the immunoconcentration of uterine myometrial scar collagen type VI, elastin, smooth muscle myosin heavy chain, alpha-smooth muscle actin, and endothelial cell marker cluster of differentiation 31 remains consistent regardless of the duration elapsed since the previous CS. The findings indicate that there are no significant alterations in the biomechanical properties of the uterine muscle beyond a period of 13 months following a CS.

## 1. Introduction

In recent years, the cesarean section (CS) has emerged as the most commonly performed major surgical procedure, with a notable increase in its occurrence. In numerous instances, pregnancies are concluded through the implementation of CS, either upon the explicit request of the individuals involved or in situations where patients have previously undergone CS as a precautionary measure due to concerns surrounding uterine rupture. The process of myometrial wound healing plays a crucial role in determining the future morphology and functional behavior of uterine muscle, as well as the associated risk of uterine rupture in subsequent pregnancies [1,2].

### 1.1. The Wound Healing Process

Wound healing is a physiological reparative mechanism that can be influenced by various factors, potentially resulting in impaired wound healing. There are certain factors that can influence wound characteristics at the local level, including oxygenation, infection, and vascularity. Conversely, there are systemic factors that can impact healing ability, such as diabetes, obesity, stress, age, sex hormones, and smoking. The process of normal wound healing is associated with a fibroproliferative process involving several mediators as well as the involvement of blood cells and extracellular matrix parenchymal cells. The aforementioned procedure consists of three distinct stages: inflammation, tissue formation, and tissue maturation and remodeling. The inflammation phase spans from the initial occurrence of injury until approximately 4–6 days thereafter. This is followed by the tissue formation phase, which occurs between days 4 and 14. Finally, the tissue maturation and remodeling phase takes place from week 1 to year 1. There is currently insufficient evidence to support the notion that the optimal myometrialstructure and functional integrity, which leads to a reduced risk of uterine scar rupture in subsequent pregnancies, can be achieved within a 24-month period following a CS [3,4]. Consequently, we opted to investigate the integrity of the cluster of differentiation 31 (CD31) antigen, which is widely regarded as a highly reliable marker for evaluating tissue density [5]. 

### 1.2. The Cluster of Differentiation 31 Antigen

It is a single-chain transmembrane glycoprotein expressed not only on the surface of the platelets but also in granulocytes, endothelium, and monocytes. The CD31 molecule has an extracellular domain, which contains six homology units of the C2 subclass, typically molecules with the option to adhere to different cells. The CD31 protein plays a crucial role in the physiological mechanism of wound healing. Because of its inherent characteristics, this particular antigen holds significant importance in evaluating the level of vascular maturity and stabilization of the endothelial cell monolayer [4,6]. There are some indications that the CD31 antigen is not only involved in wound healing but also in interactive events during thrombosis and angiogenesis [2,6].

### 1.3. Structural Proteins in the Repair and Healing Process

Collagens and elastin, classified as structural proteins, are highly prevalent and integral components of the extracellular matrix (ECM). These proteins play a variety of critical roles, including facilitating cell adhesion and migration, contributing to tissue morphogenesis, and participating in the creation of tissue scaffolds. Some findings emphasize the role of collagen type VI in differentiation and embryonic development. It was also found in the scar tissue of advanced-age wound lesions.

Additionally, they actively engage in the process of repairing and facilitating healing. The basement membrane, a significant component of the extracellular matrix (ECM), serves essential functions in providing mechanical support, maintaining homeostasis, and facilitating repair processes. Type VI collagen, a component of microfibrillar structures located near blood vessels and nerves, is a distinctive constituent of basement membranes (BM), comprising three alpha chains (VI): α1, α2, and α3. Following its secretion into the extracellular space, it undergoes a process of network formation, which serves to facilitate the establishment of a molecular scaffold and enable interactions with other components of the basement membrane. Elastin is a polymeric protein found in connective tissue that provides elasticity to elastic tissues in vertebrates. The alterations in collagen and morphological properties of elastin fibers within the extracellular matrix (ECM) may potentially be impacted by aberrant healing and pathological mechanisms, ultimately resulting in tissue fibrosis and atherosclerosis. The role of collagen type VI is cell migration [2,7,8]. 

The quantification of alpha-smooth muscle actin (aSMA) levels in the uterine scar may serve as an indicator of biomechanical muscle properties. This marker, when exposed to proinflammatory cytokines (IFN-γ), exhibits a notable decrease in tonic contractility. Under typical circumstances, when muscles undergo adaptation to either longer or shorter lengths, there is an observed fluctuation in the recruitment of myosin monomers and oligomers into the actin filament lattice. Cytoplasmic actin is part of the microfilament system of cytoskeletal proteins. Myosin is a contractile-muscle-specific protein composed of two heavy and four light chains. The myosin heavy chain has many isoforms; they are specific for different muscle or fiber types, some of which are developmentally regulated. Smooth muscle myosin heavy chain (SM-MHC) is a cytoplasmic structural protein that is a major component of the contractile apparatus in smooth muscle cells. It has been reported to be specific to smooth muscle development. Its dysfunction may occur in conditions of scarred smooth muscle. There is a potential for alterations in the levels of smooth muscle myosin heavy chain (SMMhc) in cases involving a scarred lower uterine segment [9]. 

It is hypothesized that the levels of collagen type VI, elastin, smooth muscle myosin heavy chain (SMMhc), alpha-smooth muscle actin (aSMA), and the endothelial cell marker CD31 in uterine CS may undergo alterations during the interpregnancy intervals between successive cesarean sections. The objective of this study was to examine the immunohistochemical characteristics of the uterine scar after surgery during various stages of healing. This was achieved by analyzing the expression and concentration of myometrial elastin, collagen type VI, aSMA, SMMhc, and the endothelial cell marker CD31 in scarred uteri. The analysis was conducted based on the interdelivery period during term gestations.

## 2. Material and Methods 

This research was carried out at the Department of Perinatology, Obstetrics, and Gynecology, Pomeranian Medical University, Szczecin, Poland. This study received approval from the Commission of Bioethics at the Pomeranian Medical University in Szczecin. Over a span of three years, our department conducted a prospective observation to examine the total number of deliveries. The findings revealed that out of the 4668 deliveries, 2395 cases (51.3%) involved the performance of cesarean sections (CS). This study comprised a sample of 177 pregnant women who met the criteria of being in good health.

Eligible participants were pregnant women meeting the following inclusion criteria: gestational age ≥ 37 weeks, uncomplicated pregnancy, current obstetric indications for termination of pregnancy by elective cesarean section, and consent to the proposed study. Women with a history of other uterine surgeries and women with known complications such as heart disease, hyperthyroidism, gestational diabetes, and gestational hypertension were excluded. Each consecutive patient who met the inclusion criteria during the study period was included in this study. These individuals willingly provided their consent for the collection of biological samples. Pregnant women typically undergo delivery during the early term period, which spans from 37 0/7 weeks to 38 6/7 weeks of gestation. Additionally, delivery commonly occurs during the full term period, which encompasses the timeframe from 39 0/7 weeks to 40 6/7 weeks of gestation. Out of the total sample size, 52 individuals (29.4%) experienced their initial pregnancy, while 125 individuals (70.6%) had undergone a cesarean section in the past. 

The average neonatal weight across all groups was 3271 ± 0.528 g. There were no statistically significant differences observed in maternal age, maternal weight, gestational age, and newborn body weight between the seven groups (Table 1). The trial size for the population of 100,000 was determined by estimating the proportion size in the range of 4–5%. This study employed a confidence level of 95% and an acceptable error margin of 10%, resulting in a sample size of either 15 or 18 women in each group.

Based on the duration elapsed since the previous cesarean section, women were categorized into the following seven distinct groups:

- Group 1 consisted of 52 primipara participants.
- Group 2 consisted of 15 participants, 13–23 months.
- Group 3 consisted of 17 participants, 24–30 months.
- Group 4 consisted of 21 participants, 31–36 months.
- Group 5 consisted of 16 participants, 37–42 months.
- Group 6 consisted of 29 participants, 43–60 months.
- Group 7 consisted of 27 participants, more than 60 months.

None of the pregnant women who were evaluated were in labor, and there were no identifiable factors that could potentially impact the process of myometrial healing. None of the pregnant individuals exhibited any risk factors associated with abnormal wound healing, such as smoking, gestational diabetes, hypertension, or other comorbidities. Table 1 presents the clinical characteristics of the women who participated in this study and underwent cesarean delivery.

### 2.1. Reagents Used for Immunohistochemical Determinations

Novocastra^TM^ IHC Antibodies, Probes & Leica BOND reagents were used for immunohistochemical determinations: Elastin (Novocastra Elastin Clone BA-4 0.5 mL lyophilized NCL-ELASTIN P (enzyme)); Collagen VI (Novocastra Collagen Type VI (D3 Chain) Clone 64C11 1 mL lyophilized NCL-COLL-VI P (HIER)); Cd31 (Novocastra CD31 (PECAM-1) Clone 1A10 1 mL, 0.1 mL lyophilized NCL-CD31-1A10 P (HIER) 7 mL BOND ready-to-use PA0250 P (HIER)); aSMA (Novocastra Alpha-Smooth Muscle Actin (SMA) Clone Dsm-1 1 mL lyophilized NCL-SMA F P (enzyme) W 7 mL ready-to-use RTU-SMA F P (enzyme) 7 mL BOND ready-to-use PA0943 P); SMMhc (Novocastra Myosin Heavy Chain Antibodies Clone S131 1 mL, 0.1 mL lyophilized Myosin Heavy Chain (smooth muscle) NCL-MHC-Sm F P (HIER) 7 mL BOND ready-to-use PA0493 P (HIER)).

### 2.2. Surgical Procedures 

All patients underwent surgical procedures while under the administration of epidural anesthesia. The experimental procedure was conducted under aseptic conditions. A Pfannenstiel skin incision was performed, extending to the subcutaneous fascial layer. The fascia was surgically cut along the midline and then extended in a lateral direction. Upon the initiation of the abdominal cavity incision, a transverse cut was made on the lower uterine segment. The newborn was delivered without any trauma. Following the delivery of the newborns, the lower segment of the uterus, which had been scarred, was accurately identified as an irregular formation of scar tissue. Subsequently, a tissue sample in the rhomboid shape, the diagonals of which were about 20 mm, was surgically removed and collected. Based on the available evidence derived from randomized trials, which failed to provide conclusive support for a particular method of uterine closure to achieve optimal maternal outcomes, our department employed a one-layer closure technique utilizing continuous lock stitches for closing the uterine incision. A hysterectomy was not deemed necessary, and there were no reported cases of maternal or neonatal mortality. 

**Figure 1:**
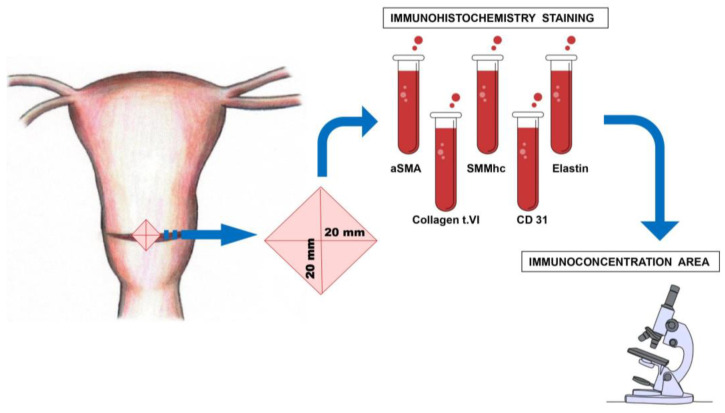
The location and method of the myometrial sample excision, taken from the lower uterine segment during the cesarean section.

The location and method of excision of the uterine scar fragment are shown in Figure 1.

### 2.3. Morphological Study 

The biopsies collected from the patients were fixed in a 4% buffered formalin solution. The tissues were prepared for analysis by fixing them in formalin and embedding them in paraffin. Subsequently, the tissues were sliced into sections using a microtome, with each section measuring approximately 4–5 μm in thickness. Subsequently, the aforementioned sections were affixed onto glass slides that had been coated with poly-L-lysine. In order to conduct morphological analysis, the slides were stained with hematoxylin–eosin (H-E9).

### 2.4. Immunohistochemistry 

Immunohistochemistry (IHC) is a commonly employed technique in the field of biomedical research for the identification of specific protein markers, including CD31 (PECAM-1), a platelet endothelial cell adhesion molecule. Additionally, IHC is utilized to detect α-actin and the myosin heavy chain, which are integral components of myofilaments found in smooth muscle cells. Furthermore, IHC is employed to identify elastin and collagen type VI, both of which are constituents of the extracellular matrix. In order to visualize the proteins present in the myometrium scar, a set of mouse anti-human antibodies (Novocastra^TM^ IHC Antibodies, Probes & Leica BOND) was used. The antibodies used included anti-CD31 (clone 1A10; diluted at 1:50, with primary antibody incubation at 25 °C), anti-smooth muscle actin, alpha (clone ASM-1; diluted at 1:50, with primary antibody incubation at 25 °C), anti-myosin heavy chain (smooth muscle) (clone S131; diluted at 1:25, with primary antibody incubation at 25 °C), anti-elastin (clone BA-4; diluted at 1:100, with primary antibody incubation at 25 °C), and anti-collagen type VI (clone 64C11; diluted at 1:50, with primary antibody incubation at 25 °C). The sections of myometrium scar that had been deparaffinized were subjected to epitope retrieval through heat induction, using citrate buffer with a pH of 6.0, by microwaves. Additionally, a gradual cooling process was implemented to reach room temperature, followed by a thorough washing of the slides in phosphate-buffered saline (PBS) for a duration of five minutes, repeated twice. Subsequently, the slides were incubated for a period of 60 min with primary antibodies. The subsequent sections were subjected to staining using the avidin–biotin–peroxidase system, with diaminobenzidine serving as the chromogen. This staining procedure was performed in accordance with the instructions provided in the Dako EnVision+System manual, specifically using the EnVision+System-HRP (DAB) kit with the code K4010 from DakoCytomation, located in Glostrup, Denmark. The sections were rinsed with distilled water and subsequently stained with hematoxylin as a counterstain. To establish a negative control, the specimens were subjected to processing without the presence of the primary antibody. In this case, the primary antibody was substituted with non-immune mouse serum. Positive staining was determined through the utilization of a microscope, whereby the identification of visual brown pigmentation was employed.

The slides underwent analysis using an Olympus BX 46 light microscope and an Olympus DP 25 camera. CD31, also known as platelet endothelial cell adhesion molecule (PECAM-1), exhibits staining properties not limited to endothelial cells within blood vessels but also demonstrates sensitivity and efficacy in identifying such cells. Endothelial cells from lymphatic vessels can also be stained, albeit to a lesser extent compared to endothelial cells from arteries and veins. In the course of our inquiry, we refrained from employing a lymphatic-specific marker such as LYVE1 or D240, as these markers have been observed to exhibit staining capabilities not limited to lymphatic cells but also encompassing other cell types, including mesothelial cells and myoepithelial cells. H&E staining allowed us to differentiate larger-caliber arterioles and venules from lymphatics based on their distinct anatomical features. Specifically, the arterioles and venules exhibited thicker walls in comparison to the lymphatic vessels, which possessed thin walls accompanied by a thin layer of basement membrane. Furthermore, the lymphatic vessels were surrounded by collagen and lacked erythrocytes within their luminal space. Veins and capillaries, characterized by their thin walls, exhibited a resemblance to the lymphatic system in terms of their contour.

### 2.5. Digital Image Analysis (Digital Computer-Assisted Analysis Technique) 

The stained slides underwent scanning at a magnification of 20× objective using high-content screening, enabling efficient quantification and comparison of data across various samples. The analysis technique employed in this study utilized a digital computer-assisted approach. Specifically, an image processing program called Cell Sense Dimension 1.5, developed by Olympus, was utilized. The primary objective of this technique was to identify and locate regions within the images that exhibited immunohistochemical staining for CD31, aSMA, SMMhc, elastin, and collagen type VI. The data acquired for each group were presented in the form of mean area measurements in square micrometers (μm^2^) and expression index, expressed as the percentage of immunopositive staining concentration.

### 2.6. Statistical Analysis

In order to select the appropriate statistical analysis, we conducted a Shapiro–Wilk normality test to assess the normal distribution of the dependent variables. Due to the lack of a normal distribution in our data, we employed a nonparametric Mann–Whitney U test to assess the disparities among the examined groups. We conducted a comparison of the mean and median scores of the samples and subsequently performed a one-way analysis of variance using the Statistica10 statistical software. A significance level of *p* ≤ 0.05 is employed as the threshold for determining statistical significance.

## 3. Results 

**Figure 2:**
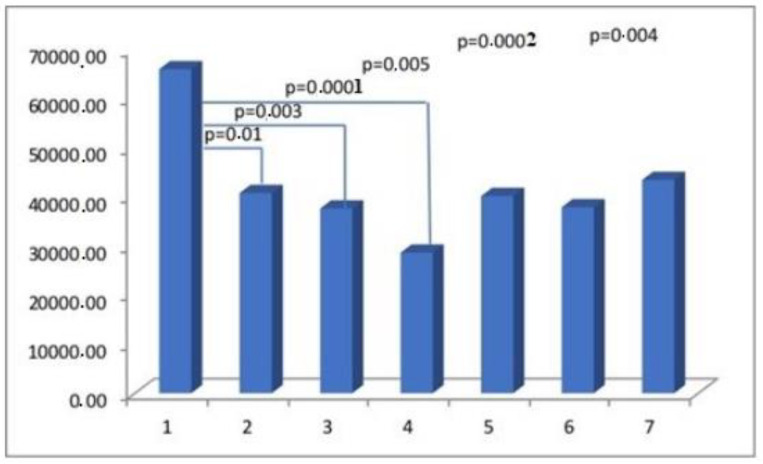
The myometrial immunoconcentration of alpha-smooth muscle actin was analyzed in the different groups, and the results were measured in square micrometers (μm^2^).

**Figure 3:**
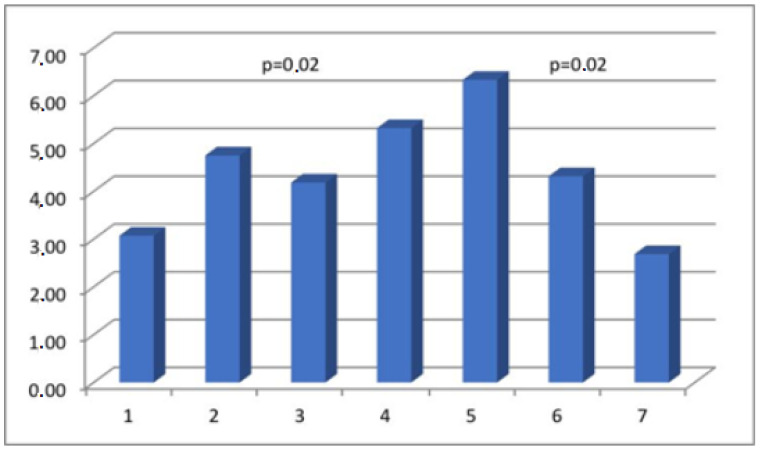
The myometrial immunoconcentration of smooth muscle myosin heavy chain was analyzed in the different groups, and the results were measured in square micrometers (μm^2^).

**Figure 4:**
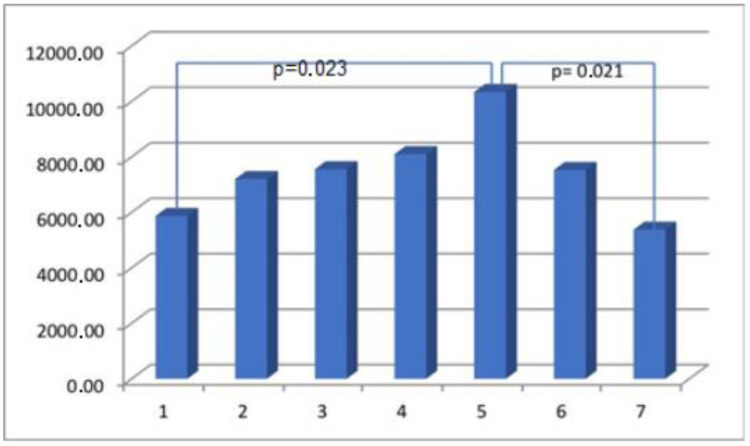
The myometrial immunoconcentration of collagen type VI was analyzed in the different groups, and the results were measured in square micrometers (μm^2^).

**Figure 5:**
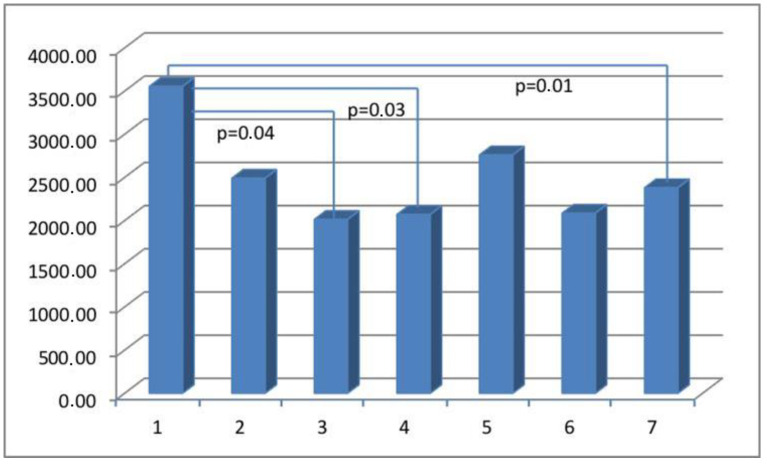
The myometrial immunoconcentration of the endothelial cell marker CD31 was analyzed in the different groups, and the results are measured in square micrometers (μm^2^).

**Figure 6:**
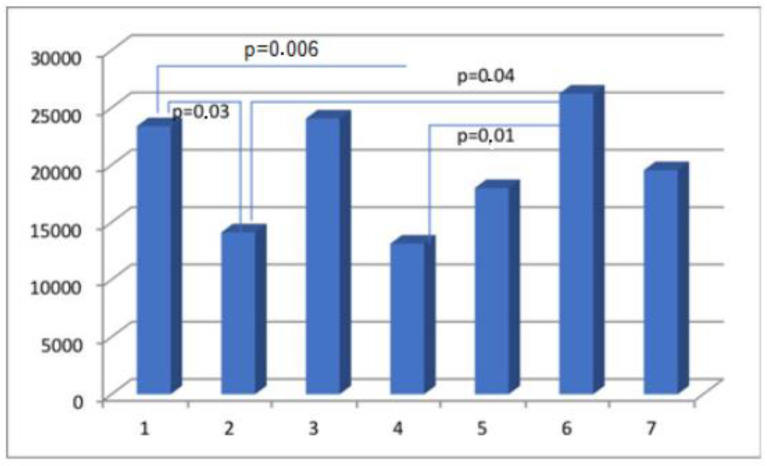
The percentage of immunopositive myosin-staining cells in scar tissue in the analyzed groups.

The results obtained from the semi-quantitative evaluation using immunohistochemical methodology did not align with the anticipated outcomes. Our intention was to demonstrate substantial variations in the composition of scar tissue among different cohorts. Nevertheless, notable disparities were observed in the levels of actin and CD31 protein between Group I, consisting of primipara individuals with unscarred uteri, and the remaining groups who had undergone cesarean section procedures. The presence of CD31 was detected in the endothelial cells of blood vessels, manifesting as a faint line within the lumen. The cytoplasm of myometrial smooth muscle cells (SMC) exhibited immunoreactivity for actin and myosin. Furthermore, actin was also detected in the smooth muscle cells of blood vessels. The differences in myosin and collagen concentrations among the analyzed groups were not clearly understood, while the immunoexpression/immunoconcentration of elastin did not exhibit significant changes between the groups, as shown in Table 2. Figure 2, Figure 3, Figure 4 and Figure 5 display statistically significant differences, as indicated by the calculated probability value (*p*). Subsequently, an endeavor was made to elucidate the extant disparities and connections among the groups by computing the concentration of immunopositive staining cells as a percentage (Table 3). The analysis that was performed revealed the significance of the mutual relationship, providing clear evidence that notable distinctions were observed solely between the initial group (primipara) and the remaining groups in all assessed variables, with the exception of myosin, as illustrated in Figure 6.

**Figure 7:**
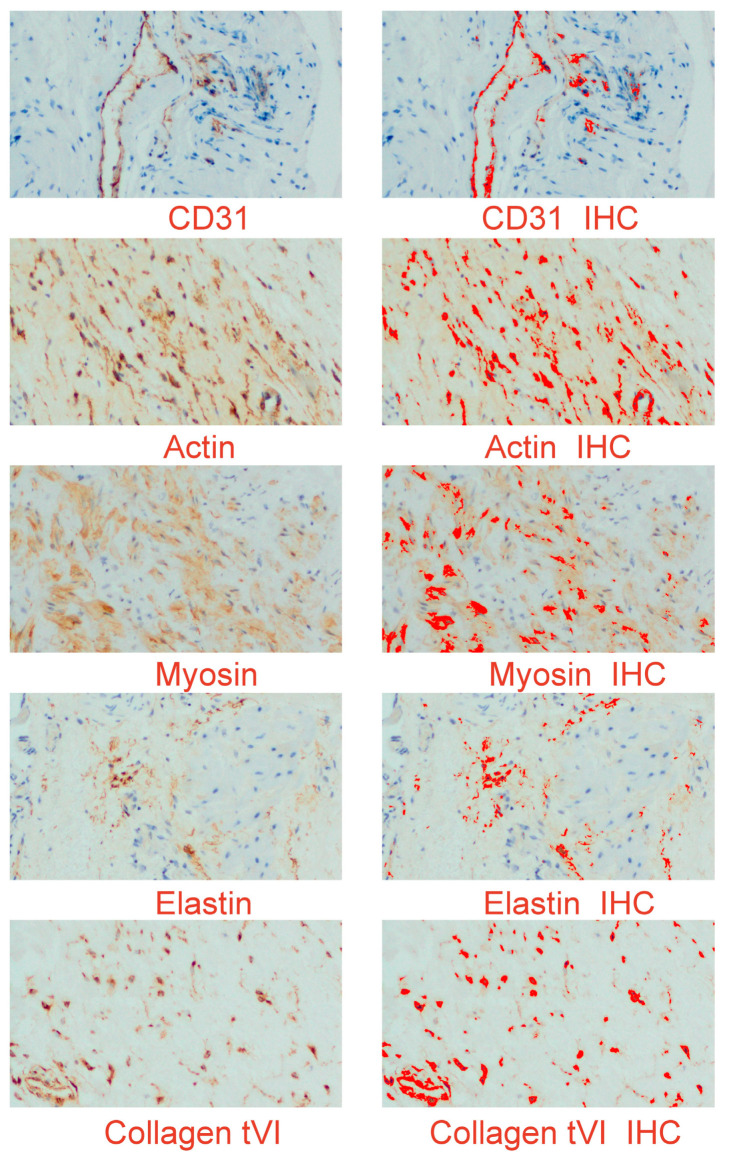
An example of digital images of the myometrial distribution of elastin, collagen type VI, alpha-smooth muscle actin, smooth muscle myosin heavy chain, and endothelial cell marker CD31 (cluster of differentiation). The left panel presents the immunohistochemical expression of the studied proteins; the right panel is a digital image, in which the product of the reaction is shown in strong red. IHC (immunohistochemistry); original objective magnification: 20×.

The objective of this study is to illustrate the favorable staining patterns observed in immunohistochemistry, which allow for the visualization and quantification of a particular antigen–antibody complex within a tissue section. This is achieved through the utilization of chromogen and microscopy techniques. The assessment of staining outcomes in immunohistochemistry (IHC) and the subsequent assignment of a grade or score represent a critical component within the overall workflow. No significant differences were observed in the evaluation of the immunohistochemical (IHC) reaction of the preparations, suggesting that the analyzed scar promoters have a consistent content regardless of the time function (Figure 7).

## 4. Discussion 

The process of wound healing holds significant biological importance, as it plays a crucial role in preserving the integrity and optimal functioning of organs. The molecular mechanisms underlying this process are still under investigation [10,11,12]. The process of wound healing consists of four distinct phases: hemostasis, inflammation, proliferation, and remodeling. Hemostasis involves the constriction of blood vessels, the aggregation of platelets, the release of granules, and fibrin formation. Inflammation is characterized by the infiltration of neutrophils, monocytes that differentiate into macrophages, and lymphocytes. The proliferation phase involves re-epithelialization, angiogenesis, and collagen synthesis. Lastly, the remodeling phase includes collagen remodeling, maturation of blood vessels, and regression. The process of mesenchymal fibroblasts transitioning into myofibroblasts is a significant factor in the remodeling of connective tissue [4]. Alpha-smooth muscle actin (α-SMA) is a marker that is indicative of fully differentiated myofibroblasts. The histopathological analysis of the myometrium tissues that were injured provided evidence of modified healing processes, characterized by the presence of myofiber disarray, elastosis, tissue edema, and inflammation [13,14]. Myofiber disarray and elastosis have been identified as potential indicators of abnormal wound healing after iatrogenic uterine trauma [15]. During the course of our investigation, it was observed that there were no significant variations over time in the proportions of elastin, collagen type VI, SMMhc, and the endothelial cell marker CD31 expressed in the scarred lower uterine segments. Collagen is postulated to be a crucial constituent of muscular tissue, exerting a significant impact on its biomechanical properties. The uterine wall contains various types of collagen, primarily types I, III, V, and VI. Type VI collagen is found in high quantities within the endometrium, particularly during the secretory phase. Subsequently, its concentration decreases. In the myometrium, collagen type VI coexists with other collagen types and forms a surrounding matrix that interacts with smooth muscle cells. The presence of mutations in human collagen type VI within the field of pathology has been observed to result in various clinical manifestations, including muscular dystrophy, joint hyperlaxity, and contractures. The presence of elevated levels of type VI collagen in a live model of wound healing following myocardial infarction has been observed to have an impact on muscle function and remodeling during the days to weeks following the injury [16]. The orientation of collagen network fibers can be modified by labor and scarring as well. The myometrium of the lower uterine segment that has been scarred exhibits a greater concentration of collagen in comparison to unscarred myometrium samples obtained during labor [17]. Uterine dehiscence cases exhibit elevated collagen levels in the scarred lower uterine segments, potentially indicating modified biochemical dynamics during the scarring process [18]. The findings of our study indicate that there were no statistically significant variations in the immunoconcentration of collagen type VI in the uterine scars within the timeframe of 12 to 186 months following a CS. The mechanical characteristics of tissue are determined by the extracellular matrix (ECM) proteins, with fibrillary collagen [19,20] being the primary component. Additional significant components comprise proteoglycans, hyaluronate, elastin, and water. It is widely recognized that the human uterus undergoes a significant increase in wet weight, collagen content, and elastin content during pregnancy, with an approximate 11-fold increase in wet weight, a 7-fold increase in collagen content, and a 5-fold increase in elastin content [20]. Previous research on the reproductive tract in both human and animal models has demonstrated significant changes in the composition of collagen, elastin, and other extracellular matrix (ECM) proteins in the uterus and vaginal wall during gestation [21,22,23]. During the process of rapid uterine involution following parturition, which is approximately 75% complete within 8–11 days after delivery, there is a rapid breakdown of elastin and collagen [24,25].

Women who have had six or more pregnancies have twice the amount of collagen and five times the amount of elastin compared to nulliparous women [21]. There appears to be a potential correlation between the reduced expression of elastin and the severity of pelvic organ prolapse in women, suggesting that elastin may have a specific role in the development of this dysfunction [26]. 

In the course of our investigation, no discernible variations in elastin content were observed in the scarred uteri, irrespective of the length of the interpregnancy interval. This implies that the tensile scar properties exhibit no alteration subsequent to the surgical procedure.

The potential mechanisms underlying the early formation of scars in wounds encompass the activation of fibroblasts, the acquisition of alpha-smooth muscle actin (aSMA), its expression, and subsequent transformation into myofibroblasts. The myofibroblasts are responsible for the synthesis and deposition of extracellular matrix (ECM) components. They also possess contractile properties, which are attributed to the expression of alpha-smooth muscle actin (aSMA) in microfilament bundles or stress fibers. These cellular characteristics play a significant role in the contraction and maturation of the granulation tissue [27]. Immunohistochemical staining for alpha-smooth muscle actin (aSMA), widely regarded as the most reliable marker of the myofibroblastic phenotype, was therefore used to determine the concentration of myofibroblasts in scar tissue. The detection of alpha-smooth muscle actin (aSMA) within the examined uterine scars may serve as evidence for the presence of an incompletely healed wound. In addition to their expression of myofibroblast-specific markers, myofibroblasts have the capacity to express alternative contractile proteins, including the smooth muscle myosin heavy chain (SMMhc) and desmin [28]. Nevertheless, no significant variations were observed in the immunoconcentration of aSMA and SMMhc among the groups under investigation (Figure 2).

Angiogenesis is a crucial physiological process in wound healing, and the establishment of a functional vascular network is of significant importance in this regard [29,30,31,32]. Angiogenesis likely plays a role in facilitating the transportation of oxygen and nutrients to the site of injury, which is essential to supporting the rapid proliferation of reparative cells [3,33]. The angiogenic effect can be augmented by sex steroids [11]. Despite extensive research on the relationship between angiogenesis and wound repair, the extent to which angiogenesis contributes to the healing process under typical conditions remains uncertain [4,10,29]. The identification of CD31 antigen within the examined cesarean uterine scars implies its potential involvement in interactive processes associated with angiogenesis and wound healing, owing to its specific properties. No significant changes in CD31 scar immunoexpression were observed in relation to the interdelivery interval period. However, notable differences were observed in relation to primipara, as depicted in Figure 5. Hence, we focus our attention on notable disparities in the composition of the uterine myometrium between primiparous women and those who have undergone CS, irrespective of the timing of the postponed surgical procedure. While the occurrence and outcomes of uterine rupture in women who have undergone prior CS are more commonly observed, there is a lack of available data regarding the potential for prevention [34,35,36,37,38,39]. There is limited evidence regarding the effect of the number of previous cesarean deliveries, the length of time between deliveries, or the type of previous uterine scar on cesarean scar characteristics [40,41,42,43,44]. According to the guidelines provided by the Royal College of Obstetricians and Gynaecologists, a time interval of less than 2 years between a previous cesarean birth and a subsequent vaginal birth after CS can be considered a contributing factor that is associated with a reduced probability of planned vaginal births after CS. Insufficient data exist regarding the potential elevation of uterine rupture risk in women who have undergone prior myomectomy or have experienced a short interdelivery interval of less than two years [45,46,47,48,49].

In summary, our findings demonstrate that there is no apparent impact on the immunohistochemical composition of the scar when a longer interval is observed between successive CS deliveries. Our findings indicate that there is no significant alteration in the concentrations of uterine cesarean myometrial scar collagen type VI, SMMhc, aSMA, elastin, and endothelial cell marker CD31 over time following a previous CS. The findings indicate that there are no significant alterations in the biomechanical properties of uterine muscle beyond a 13-month period following a CS. These recommendations necessitate the meticulous implementation of controlled clinical trials pertaining to this subject matter.

## Figures and Tables

**Table 1:** The clinical characteristics of women who have undergone cesarean section deliveries, with a focus on the length of the interpregnancy interval.

	Interpregnancy Interval (Months)	Maternal Age (Years) *	Maternal Weight (kg) *	Gestational Age (Weeks) *	Birth Weight (kg) *
Group I	-	28.05 ± 3.71	75.04 ± 8.09	38.6 ± 1.2	3.256 ± 0.556
Group II	20.07 ± 3.03	32.5 ± 4.46	82.45 ± 18.61	38.67 ± 0.98	3.354 ± 0.279
Group III	26.35 ± 2.45	31.27 ± 4.13	78.63 ± 12.62	38.42 ± 1.52	3.318 ± 0.674
Group IV	34.75 ± 1.29	34.81 ± 5.0	81.21 ± 14.57	37.95 ± 2.85	3.281 ± 0.683
Group V	44.25 ± 3.39	33.71 ± 7.12	81.39 ± 15.92	38.06 ± 2.14	3.215 ± 0.568
Group VI	54.69 ± 5.11	33.36 ± 4.56	80.61 ± 12.36	38.14 ± 1.15	3.334 ± 0.328
Group VII	92.61 ± 33.10	32.82 ± 4.83	81.96 ± 11.63	37.93 ± 2.1	3.231 ± 0.656

* the value of *p* ≤ 0.05 is the cutoff for statistical significance; no statistically significant differences were found between the groups.

**Table 2:** The groups were analyzed for the immunoconcentration of elastin, collagen type VI, alpha-smooth muscle actin (aSMA), smooth muscle myosin heavy chain (SMMhc), and the endothelial cell markers CD31 (cluster of differentiation) or PECAM-1 (platelet and endothelial cell adhesion molecule-1) in the myometrium. The immunoconcentration values were measured in square micrometers (μm^2^).

Parameter	Actin Area (aSMA)	Myosin Area (SMMhc)	Elastin Area	Collagen Area (Collagen t.VI)	CD 31 Area (PECAM-1)
Group I	65,933.65 ± 33,522.77	5870.08 ± 5939.30	5002.57 ± 3823.45	23,379.68 ± 14,945.39	3553.13 ± 2932.26
Group II	40,730.74 ± 27,791.32	7198.77 ± 6749.24	5522.69 ± 6390.30	14,122.49 ± 11,119.03	2490.21 ± 1523.59
Group III	37,578.51 ± 33,305.28	7554.65 ± 8615.21	4972.82 ± 4437.13	24,045.47 ± 21,182.52	2013.35 ± 1587.92
Group IV	28,598.35 ± 23,383.7	8097.26 ± 8845.94	3805.66 ± 4234.11	13,136.13 ± 11,771.61	2070.71 ± 1417.69
Group V	40,118.16 ± 21,442.87	10,338.99 ± 9192.84	7909.23 ± 9559.51	17,991.79 ± 13,068.37	2760.05 ± 2155.23
Group VI	37,833.75 ± 26,360.83	7569.92 ± 6656.56	9693.78 ± 17235.27	26,225.81 ± 20,777.57	2084.36 ± 1282.20
Group VII	43,394.51 ± 30,965.22	4837.22 ± 6096.49	5604.20 ± 5449.66	19,540.48 ± 15,068.75	2380.13 ± 1564.63

**Table 3:** Myometrial immunoconcentration of elastin, collagen type VI, alpha-smooth muscle actin (aSMA), smooth muscle myosin heavy chain (SMMhc), endothelial cell marker CD31 (cluster of differentiation), or PECAM-1 (platelet and endothelial cell adhesion molecule-1) in analyzed groups (%).

Parameter	Actin % (aSMA)	Myosin % (SMMhc)	Elastin %	Collagen % (Collagen t.VI)	CD 31% (PECAM-1)
Group I	41.33 ± 14.68	3.07 ± 4.3	2.11 ± 3.19	14.65 ± 10.91	2.06 ± 1.21
Group II	29.92 ± 19.23	4.75 ± 4.93	3.59 ± 4.64	9.94 ± 7.83	1.54 ± 0.83
Group III	21.72 ± 17.8	4.18 ± 5.16	3.19 ± 3.31	12.63 ± 8.14	1.10 ± 0.67
Group IV	19.28 ± 16.38	5.32 ± 5.64	2.27 ± 3.17	9.05 ± 8.43	1.34 ± 1.0
Group V	24.88 ± 15.83	6.33 ± 6.16	2.74 ± 3.64	12.12 ± 9.62	1.45 ± 0.79
Group VI	23.77 ± 17.43	4.32 ± 3.06	4.39 ± 4.78	13.76 ± 9.30	1.24 ± 0.8
Group VII	30.16 ± 22.2	2.69 ± 3.9	3.43 ± 4.07	12.49 ± 10.78	1.43 ± 1.01

## Data Availability

The data are available on request.

## Abbreviations

aSMA	Alpha-smooth muscle actin
BM	basement membranes
CD31	cluster of differentiation 31
CS	cesarean section
ECM	extracellular matrix
IHC	immunohistochemistry
SMC	smooth muscle cells
SMMhc	smooth muscle myosin heavy chain
